# Does Prior Experience Matter? Intention to Undergo Cervical Cancer Screening among Rural Women in South-Central Ethiopia

**DOI:** 10.3390/curroncol31090363

**Published:** 2024-08-24

**Authors:** Bezawit Ketema, Adamu Addissie, Sarah Negash, Eva Johanna Kantelhardt, Mirgissa Kaba

**Affiliations:** 1 School of Public Health, College of Health Sciences, Addis Ababa University, Addis Ababa, Ethiopia; 2 Institute for Medical Epidemiology, Biostatistics and Informatics, Martin-Luther-University, 06097 Halle, Germany; 3 Global Health Working Group, Martin-Luther-University, 06097 Halle, Germany

**Keywords:** cervical cancer screening, rural women, primary healthcare, intention, perceived behavioural control, previous screening experience, Ethiopia

## Abstract

Early screening for cervical cancer has substantially reduced the morbidity and mortality attributed to it. This study aimed to assess factors that affect the intention to undergo cervical cancer screening among rural women attending primary healthcare facilities in south-central Ethiopia. A health-facility-based, cross-sectional study design was employed for which the calculated required sample size was 427. An interviewer-administered structured questionnaire was adapted from previously published research and used to collect data. Statistical Package for Social Sciences (SPSS) version 27 was used for the statistical analysis. A logistic regression model was used to determine the factors that influenced the women’s intention to undergo cervical cancer screening. A total of 420 women participated in this study, with a response rate of 98%. The mean score from the questionnaire that was used to assess the women’s intention to undergo cervical cancer screening was 10.25 (SD ± 2.34; min 3, max 15). The absence of previous screening experience (AOR: 0.498; 95% CI 0.27–0.92) and high degree of perceived behavioural control (AOR, 0.823; 95% CI 0.728–0.930) were significantly negatively associated with women’s intention to undergo cervical cancer screening. Previous screening experience and perceived behavioural control significantly influenced the intention to undergo cervical cancer screening. Women in rural areas could, therefore, benefit from awareness-creation programmes that focus on these factors.

## 1. Introduction

Approximately 90% of global cervical cancer deaths in 2020 occurred in low- and middle-income countries [1]. In Ethiopia in 2019, 1.6% of total deaths in women were caused by cervical cancer [2].

Cervical cancer can be prevented by effective approaches such as HPV (Human Papillomavirus) vaccination, cervical lesion screening, and the treatment of precancerous lesions [3,4]. However, an estimated 95% of women in developing countries have never been screened for cervical cancer [5]. Most women in developing countries are diagnosed with cervical cancer when they have advanced disease [6,7]; this is related to a poor prognosis as advanced cervical cancer has a lower probability of being cured compared with cervical cancer that is treated at an earlier stage [8].

The government of Ethiopia planned to achieve 80% coverage for cervical cancer screening (CCS) among women aged 30–49 by 2020 [9]. Despite this plan, only approximately 28% (160,290) of the planned number (578,778) were screened for cervical cancer in 2020/2021, which equated to 1.2% of the eligible women in the country [10].

Existing evidence suggests that there is a low level of intent to undergo CCS in Ethiopia [11,12]. Studies conducted in various countries, including Ethiopia, have shown that the intention to undergo CCS can be affected by various personal and community-level factors; such factors include educational status [13,14], monthly income, age [15], previous screening practice [16], HIV serostatus, HPV vaccination status [17,18], knowledge about cervical cancer, attitude to CCS, subjective norm, and perceived behavioural control [11,12,18,19,20].

Previous studies, including several that were conducted in Ethiopia, have applied the theory of Planned Behaviour (TPB) to describe ‘intention towards CCS’ behaviour [11,12,20,21,22]. TPB is one of the social cognitive theories that is most commonly used to describe relationships between human behaviour and an individual’s intention toward the behaviour. As per Ajzen’s TPB, intention is affected by favourable or unfavourable attitudes toward the behaviour, the subjective norm, and the individual’s perceived behavioural control, which results from behavioural beliefs (beliefs about the likely consequences and experiences related to the behaviour), normative beliefs (beliefs about the normative expectations and behaviours of significant others), and control beliefs (beliefs about the presence of factors that may enable or hinder the performance of this behaviour), respectively [23].

The studies that have previously described women’s intention to undergo CCS in Ethiopia were conducted in towns, and therefore did not examine the intention to undergo CCS in rural settings in Ethiopia. Thus, this study was conducted among rural women attending primary healthcare facilities in south-central Ethiopia. The findings of this study can, therefore, be used in designing interventions targeting women’s behavioural intention towards CCS in rural settings.

## 2. Materials and Methods

### 2.1. Study Design, Study Area, and Study Period

A health-facility-based, cross-sectional study design was employed for this study, which was conducted from 1 to 15 February in 2021 in the Gurage zone and Southwest Shewa zone of south-central Ethiopia. According to a population projection report from the Ethiopian Central Statistics Agency (CSA), the Gurage and Southwest Shewa zones had total populations of 1,791,034 and 1,602,719 million, respectively, in July 2021 [24]. As per the current data from the Ethiopian Ministry of Health’s website, there are 75 and 54 primary healthcare facilities, respectively, in the Gurage and Southwest Shewa zones [25].

### 2.2. Population and Sampling

Twelve primary healthcare facilities in the Gurage and Southwest Shewa zones that had started providing CCS services at least six months before the start of the data collection period were identified based on a previous preliminary assessment. According to the Ethiopian national cancer-control plan, women aged 30–49 years are eligible for CCS [9]. Thus, all women aged 30–49 who visited the selected healthcare facilities during the study period were eligible for inclusion in this study, and any women who had cervical cancer were excluded.

The required sample size for this study was calculated using OpenEpi software version 2.3. Based on a similar study conducted in Debre Berhan, Ethiopia [11], 45.3% of women aged 30–49 years had the intention to undergo CCS. A 5% margin of error and a 95% confidence level were assumed, as was a 12% non-response rate, and the required sample size was determined to be 427 women.

Based on data from the client registry reports of the healthcare facilities that were recorded one month before data collection for this study began, on average, 60 clients visited each of the relevant healthcare facilities per day. According to the CSA report [24], the proportion of these women who were aged 30–49 was around 8.9%, which gave the estimated availability of five women per healthcare facility per day. Thus, during the 15-day data collection period, a total of 900 women aged 30–49 years were expected to visit those 12 healthcare facilities; 427 of these women (the calculated sample size) were selected using a systematic random-sampling method with a skip interval of two.

### 2.3. Measurement and Data Collection

Ajzen’s TPB manual [23] and previous research works [11,22,26] were used for adapting the data collection tool used in this study. Contextual items that were used to measure indirect TPB constructs were identified by an elicitation study before the data collection tool was developed. The elicitation study consisted of 12 in-depth interviews with women who resembled the study’s target population. A total of twelve themes emerged from the in-depth interviews that were conducted for the elicitation study (Table S1).

The outcome variable (intention for CCS) was operationalized (recoded in to binary variable as ‘Yes’ and ‘No’ based on the observed mean score) by using the composite of three intention-measuring items (‘in the next 3 months, how likely is it that you expect to undergo CCS’, ‘in the next 3 months, how likely is it that you will want to be screened for cervical cancer’, and ‘how likely is that you will intend to be screened for cervical cancer in the next 3 months’) were used to determine each participant’s intention to undergo CCS. Each of the TPB constructs (attitude, subjective norm, and perceived behavioural control) were measured on a five-point Likert scale, and possible responses ranged from ‘strongly disagree’ (1) to ‘strongly agree’ (5). The following items were used to measure each participant’s direct attitude towards CCS: ‘CCS is beneficial’, ‘CCS is good’, and ‘CCS feels pleasant’. The items that were used to measure each participant’s direct subjective norm towards CCS were as follows: ‘most people who are important to me think that I should be screened for cervical cancer’, ‘people who are important to me want me to be screened for cervical cancer’, ‘it is expected of me to undergo CCS’, and ‘I feel under social pressure to undergo CCS’. The following items were used to measure direct perceived behavioural control towards CCS: ‘I am confident that I could do cervical cancer screening’, ‘for me cervical cancer screening is easy’, ‘the decision to take cervical cancer screening is beyond my control’, and ‘whether or not I take CCS is entirely up to me’ (Table S1).

Based on a suggestion from previous studies [11,26], knowledge about cervical cancer was also measured using ten items. The knowledge-assessing items that were used focussed on the risk factors, symptoms, and prevention options of cervical cancer; the mean observed score was computed and knowledge was categorised as ‘Good’ or ‘Poor’ according to whether scores were above or below the mean score, respectively.

The structured tool was translated into the local languages known as Amharic and Afan Oromo before the pre-test was conducted. Based on the findings of the pre-test, items were modified for clarity and flow of ideas. The final version of the questionnaire was structured to include ten sections: socio-demographic characteristics, knowledge, past behavioural practice, intention, and direct and indirect (attitude, subjective norm, and perceived behavioural control). The interviewer-administered structured questionnaire was used for data collection, which was conducted by twelve trained personnel using the Open Data Kit (ODK) collect platform.

### 2.4. Data Analysis

The collected data were exported from ODK to SPSS version 27 for analysis. Frequencies and percentages were computed and presented in tables. The Independent *t*-test was used to test the mean difference of attitude, subjective norm, and perceived behavioural control composite scores among women who did or did not intend to undergo CCS. A binary logistic regression model was used to identify factors that were associated with the intention to undergo CCS. Variables with *p*-values below 0.2 [27] were candidates for inclusion in the final multivariable logistic regression model; although the variables for knowledge and attitude did not satisfy this statistical criterion, they were included in the final regression model due to their importance with respect to the intention to undergo CCS [23]. Statistical significance was set at a 95% confidence level.

## 3. Results

### 3.1. Socio-Demographic Characteristics

A total of 420 participants completed the interview, yielding a response rate of 98%. The participants’ ages ranged from 30 to 49 years, with a mean age of 40.5 years (SD ± 6.17). More than 40% of the participants were followers of the Orthodox religion. Close to half (193; 46%) of the women were unable to read and write, and more than half (235; 56%) of the participants were housewives. Most of the participants (360; 85.7%) were married and the majority (224; 53.3%) were multiparas. Among the married participants (360), most (262; 62.4%) had spouses who were farmers (Table 1).

### 3.2. Knowledge about Cervical Cancer and Previous CCS Practices

Among the 420 study participants, 192 (45.7%) said that cervical cancer is treatable if detected early. In this study, 173 participants (41.2%) mentioned at least one risk factor for cervical cancer. The most common risk factor that was mentioned by participants was having multiple sexual partners (94; 22.4%), followed by having a family history of cervical cancer (81; 19.3%). More than half of the participants (260; 61.9%) revealed at least one symptom of cervical cancer. The most commonly reported symptom was a foul vaginal discharge, which was reported by 163 (38.8%) participants, and the next most common symptom was vaginal bleeding (138; 32.9%). Half of the participants (214; 50.8%) indicated that the reason for having CCS is to detect cervical cancer at an early stage. The majority (392; 93.3%) of the study participants do not know any CCS method.

Each of the knowledge item scores were summed and gave observed values ranging from 1 to 14. The mean knowledge score was 4.70 (SD ± 2.61; min 1, max 14), and few of the study participants (83; 19.8%) had ever had CCS. The majority of the participants who had ever undergone CCS had done so as a result of a health professional’s recommendation (70; 84.34%; Table 2).

### 3.3. Intention to Undergo CCS

Each of the intention item scores were summed, giving observed values ranging from 3 to 15. The mean intention score of 10.25 (SD ± 2.34; min 3, max 15) was used to dichotomise the intention variable; 195 (46.4%) of the respondents obtained scores that were greater than the mean value and were therefore considered to be intending to undergo CCS.

There was a significant difference between women who did or did not intend to undergo CCS in terms of their perceived behavioural control: the mean score for perceived behavioural control was significantly higher (*p* < 0.001) among women who did not intend to undergo CCS compared with women who did intend to undergo CCS. The mean score for attitude did not significantly differ between women who did or did not intend to undergo CCS (*p*-value 0.659), and this was also true for the mean subjective norm score (*p*-value 0.095) (Table 3).

### 3.4. Factors Associated with the Intention to Undergo CCS

Eight variables were selected for inclusion in the multivariable logistic regression model due to having a *p*-value less than 0.2 in the binary logistic regression analysis or due to being considered an important behavioural variable.

In the multivariable logistic regression analysis, the variables of previous CCS experience and perceived behavioural control were significantly associated with the intention to undergo CCS. Women who had never had CCS were 50% less likely (AOR: 0.498; 95% CI 0.27–0.92) to have the intention to undergo CCS than their counterparts who had ever had CCS. Furthermore, a unit of increase in the perceived behavioural control of participants equated to an 18% decrease (AOR: 0.823; 95% CI 0.728–0.930) in the intention to undergo CCS (Table 4).

## 4. Discussion

In this study, 46.4% of the participants intended to undergo CCS, and the mean score for the intention to undergo CCS was 10.25 (SD ± 2.34; min 3, max 15). This percentage is consistent with another study that was conducted in Ethiopia [11]; however, it is lower than that found in a study conducted in Ghana, where 82% of women reported that they intended to undergo CCS [28]. Similarly, the current finding is lower than findings reported from Taiwan [29], Indonesia [30] and the United Kingdom [31]. This variation might be due to differences in the study populations, as the latter study included HIV-positive women only, and these women may have benefited from counselling about the need for CCS during their follow-up contacts with healthcare providers. Another possible explanation relates to the current study being conducted in a rural area, as there is a lack of health information dissemination to rural Ethiopian populations, who may have limited access to media and other sources of information.

The current study showed that women who had never had CCS were 50% less likely to intend to undergo CCS than their counterparts who had had CCS. This finding is consistent with findings from studies conducted in southern Ethiopia [20] and China [32]. Additionally, a mixed-method study conducted in the United Kingdom reported a strong association between the previous engagement with screening appointments and the intention to undergo CCS [31]. This could be explained by the fact that women who have had previous screening experience could have received better information about prevention strategies, disease severity, and the advantages of screening due to their contact with healthcare providers compared with other women.

In this study, a unit of change in the perceived behavioural control of participants equated to an 18% decrease in the intention to undergo CCS. Similar findings were also reported by studies conducted in different parts of Ethiopia [11,12,20,33] and in Iran [34]. This indicates that communication interventions addressing women’s perceived behavioural control are required to bring about social and behavioural change that would increase women’s intention to undergo CCS.

Unlike in other studies—which were conducted in Yirgalem in Ethiopia in 2017 [20], and in Romania and Bulgaria in 2020 [35]—in this study, the subjective norm was not significantly associated with the intention to undergo CCS. This variation might be due to the difference in the research periods of the studies, as over time any population could change their subjective norm via direct or indirect exposure to various media.

Although the government of Ethiopia planned to screen nearly 16.5 million women by 2025 [36], only 160,290 women had been screened by 2021 [9]. Thus, to achieve this target in 2025, evidence-based communication interventions targeting behavioural change should be promoted. Educational interventions have already delivered demonstrated improvements in screening intent in African, Chinese-American, and Hispanic women [37,38,39].

Thus, the findings of this study could inform programmers of the ministry to design appropriate interventions that are contextually relevant for women in rural settings. However, we acknowledge the need for future studies that are supported by a qualitative approach for addressing barriers to CCS in Ethiopia.

### Strengths and Limitations of the Study

This study had definite strengths and limitations. The direct measures of TPB constructs were validated with indirect measures that were developed during the elicitation study to explore salient beliefs. However, as this study was a facility-based, cross-sectional study, its findings might not be representative of the general population. Furthermore, this study was unable to assess whether women who reported their intent to undergo the CCS actually did so.

## 5. Conclusions

We observed a high mean score of 10.25 (SD ± 2.34; min 3, max 15) for rural women’s intention to undergo CCS, and primary healthcare facilities in rural areas need to prepare to meet this demand. The absence of previous CCS experiences and behavioural control beliefs were significantly associated with the intention to undergo CCS. Thus, women in rural areas could benefit from awareness-creation programmes focusing on changing behavioural control beliefs by sharing the stories of women who have already undergone CCS. We recommend that researchers conduct future qualitative studies that explore barriers to CCS.

## Figures and Tables

**Table 1:** Socio-demographic characteristics of the study participants.

Variables	Variable Categories	Frequency	Percentage (%)
Religion (no. = 420)	Orthodox	171	40.7
	Muslim	154	36.7
	Protestant/catholic	95	22.6
Educational Status (no. = 420)	Secondary and above	44	10.5
	Primary	85	20.2
	Can only read and write	98	23.3
	Cannot read and write	193	46
Occupational Status (no. = 420)	Employed	185	44
	Housewife	235	56
Parity (no. = 420)	Nulliparous (No birth)	11	2.6
	Primiparous (1 birth)	9	2.1
	Multiparous (2 to 4 births)	224	53.3
	Grand multiparous (more than 4 births)	176	41.9
Marital status (no. = 420)	Married/living together	360	85.7
	Non-married ^a^	60	14.3
Spouse’s education (no. = 360)	Secondary and above	68	16.2
	Primary	80	19
	Only read and write	122	29
	Cannot read and write	90	21.4
Spouse’s occupation (no. = 360)	Employed	98	23.3
	Farmer	262	62.4

^a^ Divorced/widowed/separated/never married.

**Table 2:** Knowledge about cervical cancer and past CCS practices.

Variables	Variable Categories	Frequency (no. = 420)	Percentage (%)
Cervical cancer is treatable if detected early	No	228	54.3
	Yes	192	45.7
Risk factors for cervical cancer (multiple responses possible)	Having multiple sexual partners	94	22.4
	Family history of cervical cancer	81	19.3
	Uncircumcised sexual partner	30	7.1
	Early onset of sexual intercourse	76	18.1
	Cigarette smoking	48	11.4
Sign or symptom of cervical cancer (multiple responses possible)	Vaginal bleeding	138	32.9
	Foul vaginal discharge	163	38.8
	Pelvic/back pain	111	26.4
	Post-coital bleeding	55	13.1
CCS method you know (multiple responses possible)	VIA	10	2.4
	Pap smear	16	3.8
	HPV test	2	0.5
	‘I don’t know any’	392	93.3
Purpose of undergoing CCS (multiple responses possible)	To prevent cervical cancer	145	34.4
	To detect cervical cancer earlier	214	50.8
	To treat cervical cancer	143	34.0
	‘I don’t know’	110	26.2
Previous CCS experience	Has ever had CCS	83	19.8
	Never had CCS	337	80.2
Reasons for having CCS (no. = 83) *	Health professional’s recommendation	70	84.34
	Relative’s/friend’s recommendation	5	6.02
	Campaign or community mobilisation	5	6.02
	Television, radio, magazines, brochures	3	3.61

* Among those participants who had ever had CCS. CCS cervical cancer screening.

**Table 3:** TPB construct scores among women who did or did not intend to undergo CCS.

TPB Constructs	Intended to Undergo CCS		Not Intended to Undergo CCS		*p*-Value
	Mean	SD	Mean	SD	
Attitude	13.35	3.12	13.23	3.0	0.659
Subjective norm	12.95	3.9	12.29	4.08	0.095
Perceived behavioural control	11.77	2.05	12.55	1.95	<0.001

**Table 4:** Factors associated with the intention to undergo CCS.

Variables		Variable Categories	Intention		COR	*p*-Value	AOR	95% CI
			Intended to Undergo CCS (n)	Did not Intend to Undergo CCS (n)				
Educational Status	Secondary and above		25	19	3.604	0.058	2.714	0.846–8.707
	Primary		45	40	3.42	0.064	1.324	0.685–2.561
	Only read and write		46	52	0.955	0.328	1.527	0.814–2.863
	Cannot read and write		79	114	Ref		Ref	
Spouses’ education	Secondary and above		34	34	1.143	0.678	0.587	0.239–1.442
	Primary		44	36	1.397	0.279	1.068	0.515–2.216
	Only read and write		45	77	0.668	0.153	0.591	0.311–1.125
	Cannot read and write		42	48	Ref		Ref	
Previous CCS experience	Never had		145	192	0.498	0.005	0.498 *	**0.27–0.92**
	Ever had		50	33	Ref		Ref	
Wealth quintile	Lowest		40	44	0.826	0.537	1.002	0.440–2.28
	Second		37	47	0.716	0.280	0.733	0.317–1.69
	Middle		39	45	0.788	0.441	1.044	0.463–2.36
	Fourth		35	49	0.649	0.165	0.712	0.309–1.64
	Highest		44	40	Ref		Ref	
Knowledge	Poor		124	137	1.122	0.569	1.314	0.81–2.132
	Good		71	88	Ref		Ref	
Attitude					1.014	0.658	1.006	0.912–1.109
Subjective norm					1.042	0.095	1.043	0.972–1.12
Perceived behavioural control					0.816	<0.001	0.823 *	**0.728–0.930**

* Significant with *p* < 0.05; COR, Crude odds ratio; AOR, Adjusted odds ratio.

## Data Availability

The data presented in this study are available on request from the corresponding author.

## Supplementary Materials

The following supporting information can be downloaded at: https://www.mdpi.com/article/10.3390/curroncol31090363/s1. Table S1: TPB questionnaire towards CCS.
