# Supplementary material for: Does Prior Experience Matter? Intention to Undergo Cervical Cancer Screening among Rural Women in South-Central Ethiopia
Source: Curr Oncol. 2024 Aug 24;31(9):4908–16. doi: 10.3390/curroncol31090363 (PMC11431658; doi:10.3390/curroncol31090363)
Supplement: Supplementary file 1 [file curroncol-31-00363-s001.zip › curroncol-3128655-supplementary.pdf]

Table S1. Theory of Planned Behavior questionnaire towards cervical cancer screening.

**1. Attitude towards cervical cancer screening**								
**1.1. Direct attitude towards cervical cancer screening**								
**Sr. no.**	**Questions**	**Response categories**						
101	Cervical cancer screening is ___	Harmful	1	2	3	4	5	Beneficial
102	Cervical cancer screening is ___	Bad	1	2	3	4	5	Good
103	Performing cervical cancer screening feels ____	Unpleasant	1	2	3	4	5	Pleasant
**1.2. Indirect attitude towards cervical cancer screening**								
**1.2.1. Behavioral beliefs towards cervical cancer screening**								
104	Cervical cancer screening detect cervical cancer at early stage	Very Unlikely	1	2	3	4	5	Very Likely
105	Cervical cancer screening is painful	Very Unlikely	1	2	3	4	5	Very Likely
106	Cervical cancer screening has side effect/s	Very Unlikely	1	2	3	4	5	Very Likely
107	Cervical cancer screening invades privacy	Very Unlikely	1	2	3	4	5	Very Likely
**1.2.2. Outcome evaluation of beliefs towards cervical cancer screening**								
108	Detecting cervical cancer at early stage is	Extremely undesirable	-2	-1	0	1	2	Extremely desirable
109	Pain of cervical cancer screening is	Extremely undesirable	-2	-1	0	1	2	Extremely desirable

110	Side effect/s of cervical cancer screening is/are	Extremely undesirable	-2	-1	0	1	2	Extremely desirable
111	Invading privacy because of cervical cancer screening is	Extremely undesirable	-2	-1	0	1	2	Extremely desirable
**2. Subjective norm towards cervical cancer screening**								
**2.1. Direct subjective norm towards cervical cancer screening**								
112	Most people who are important to me think that I Should Be screened for cervical cancer	Strongly disagree	1	2	3	4	5	Strongly agree
113	I feel under social pressure to cervical cancer screening	Strongly disagree	1	2	3	4	5	Strongly agree
114	People who are important to me want me to be screened for cervical cancer	Strongly disagree	1	2	3	4	5	Strongly agree
115	It is expected of me to cervical cancer screening	Strongly disagree	1	2	3	4	5	Strongly agree
**2.2. Indirect subjective norm towards cervical cancer screening**								
**2.2.1. Normative beliefs towards cervical cancer screening**								
116	My husband thinks I should be screened for cervical cancer	Very Unlikely	-2	-1	0	1	2	Very likely
117	My family would approve of my cervical cancer screening	Very Unlikely	-2	-1	0	1	2	Very likely
118	Elder people would approve of my cervical cancer screening	Very Unlikely	-2	-1	0	1	2	Very likely
**2.2.2. Motivation to comply with normative beliefs towards cervical cancer screening**								

119	What my husband think I should do is important to me	Not at all	1	2	3	4	5	Very much
120	my family's approval of my practice is important to me	Not at all	1	2	3	4	5	Very much
121	what elder people think I should do matters to me	Not at all	1	2	3	4	5	Very much
**3. Perceived behavioral control towards cervical cancer screening**								
**3.1. Direct perceived behavioral control towards cervical cancer screening**								
122	I am confident that I could do cervical cancer screening	Strongly disagree	1	2	3	4	5	Strongly agree
123	For me cervical cancer screening is Easy	Strongly disagree	1	2	3	4	5	Strongly agree
124	The decision to take cervical cancer screening is beyond my control	Strongly disagree	1	2	3	4	5	Strongly agree
125	Whether I take cervical cancer screening or not is entirely up to me	Strongly disagree	1	2	3	4	5	Strongly agree
**3.2. Indirect perceived behavioral control towards cervical cancer screening**								
**3.2.1. Control beliefs towards cervical cancer screening**								
126	Cervical cancer screening service is easily available	Strongly disagree	1	2	3	4	5	Strongly agree
127	Cervical cancer screening is expensive	Strongly disagree	1	2	3	4	5	Strongly agree
128	I don't think I am at risk of acquiring cervical cancer	Strongly disagree	1	2	3	4	5	Strongly agree

129	I fear of positive result after cervical cancer screening	Strongly disagree	1	2	3	4	5	Strongly agree
130	Cervical cancer screening is time taking	Strongly disagree	1	2	3	4	5	Strongly agree
**3.2.2. perceived power to influence the behaviour towards cervical cancer screening**								
131	If cervical cancer screening is not easily available, i am to be screened for cervical cancer	Very Unlikely	-2	-1	0	1	2	Very likely
132	If cervical cancer screening is expensive, i am to be screened for cervical cancer	Very Unlikely	-2	-1	0	1	2	Very likely
133	Even if i think i am not at risk of acquiring cervical cancer, i am to be screened for cervical cancer	Very Unlikely	-2	-1	0	1	1	Very likely
134	Even if i have fear of positive result, i am to be screened for cervical cancer	Very Unlikely	-2	-1	0	1	1	Very likely
135	While cervical cancer screening is time taking, i am to be screened for cervical cancer	Very Unlikely	-2	-1	0	1	1	Very likely

**4. Intention to cervical cancer screening in the next 3 months**						
		Very unlikely	Unlikely	undecided	Likely	Very likely
136	in the next 3 months, how likely is it that	1	2	3	4	5

	you expect to be screened for cervical cancer					
137	in the next 3 months, how likely is it that you want to be screened for cervical cancer	1	2	3	4	5
138	How likely is that you intend to be screened for cervical cancer in the next 3 months	1	2	3	4	5
